# Supplementary material for: Parkinson’s Disease Multimodal Complex Treatment (PD-MCT): Analysis of Therapeutic Effects and Predictors for Improvement
Source: J Clin Med. 2020 Jun 16;9(6):1874. doi: 10.3390/jcm9061874 (PMC7356837; doi:10.3390/jcm9061874)
Supplement: Supplementary file 1 [file jcm-09-01874-s001.pdf]

Journal of
Clinical Medicine

Article

Parkinson's disease Multimodal Complex Treatment (PD-MCT): Analysis of Therapeutic Effects and Predictors for Improvement

Elke Hartelt [1], Raphael Scherbaum [1], Manuel Kinkel [2], Ralf Gold [1,3], Siegfried Muhlack [1] and Lars Tönges [1,3,*]

[1] Department of Neurology, St. Josef-Hospital, Ruhr-University Bochum, 44791 Bochum, Germany; elke.hartelt@rub.de (E.H.); raphael.scherbaum@rub.de (R.S.); ralf.gold@rub.de (R.G.); siegfried.muhlack@rub.de (S.M.)
[2] Psychiatrisches Gutachtenbüro, 44795 Bochum, Germany; manuel.kinkel@gmx.de
[3] Neurodegeneration Research, Protein Research Unit Ruhr (PURE), Ruhr-University Bochum, 44801 Bochum, Germany
* Correspondence: lars.toenges@rub.de

Supplementary Materials

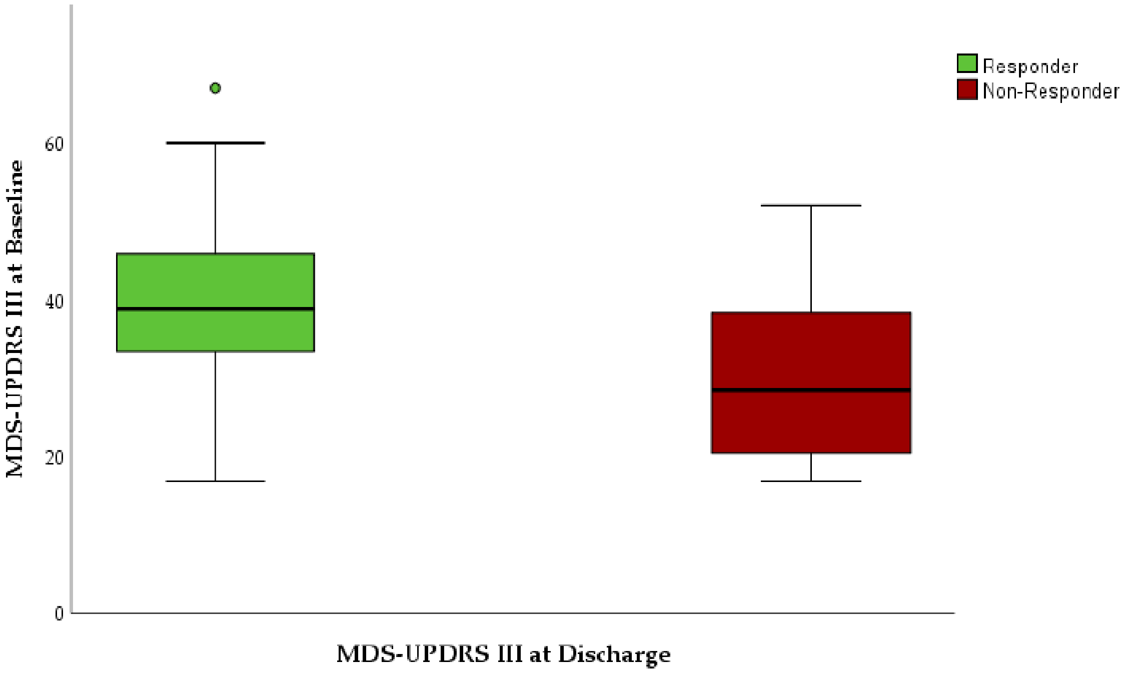

Figure S1. Comparison of MDS-UPDRS III Responders and Non-Responders. Significant mean difference in MDS-UPDRS III Responders and Non-Responders regarding BL motor level. Responders are defined as at least improvement from BL, no change is defined as Non-Responder.

Table S1. Details on the Components of Parkinson's Disease Multimodal Complex Treatment (PD-MCT) [15].

Therapy intervention	Description
Physiotherapy	- Proprioceptive training<br>- Gait analysis<br>- Posture correction<br>- Fall prevention/ balance training<br>- Amplitude training<br>- Education on self-executed home training<br>- General strengthening
Occupational Therapy	- Amplitude training (LSVT-BIG)<br>- Fine motor skill training<br>- Writing training<br>- Everyday life-oriented therapy<br>- Development of compensation strategies<br>- Fall prevention/ balance training<br>- Relaxation techniques<br>- Sensitivity and perception training
Speech and Language Therapy	- Articulation<br>- Swallowing training<br>- Language cognition<br>- Psychosocial support
Exercise/ equipment training	- General strengthening<br>- Cardiopulmonary endurance training
Physical therapy	- Massages<br>- Thermotherapy<br>- Relaxation therapy
Other	- Drug adjustment<br>- Neuropsychological assessment of emotional, cognitive and psychosocial functioning<br>- Specialized PD Nursing<br>- Social services support<br>- Advice on medial aids

Table S2. Intensity of Therapy Interventions during Parkinson's Disease Multimodal Complex Treatment (PD-MCT).

Therapy intervention	Value	SD	Min	Max
Physiotherapy [h]	4.44	0.85	1.33	5.67
Occupational therapy [h]	3.48	0.63	1.50	5.00
Speech and language therapy [h]	2.44	1.09	0.75	4.75
Exercise/equipment training [h]	3.81	1.62	0.00	7.00
Massages [h]	3.57	1.69	0.00	7.33

Amount of performed therapy hours during PD-MCT as mean value. If not indicated otherwise n=44. *h* hours, *SD* standard deviation, *min* Minimum, *max* Maximum.

Table S3. Spearman's Rank Correlation of Benefits from Baseline to Discharge in Clinical Motor Parameters with PD-MCT Therapy Hours.

Benefit variables	PT		Exercise		Physical Therapy		OT		SLT		LED_{diff}	
	r_s	p	r_s	p	r_s	p	r_s	p	r_s	p	r_s	p
MDS-UPDRS I	0.017	0.915	-0.183	0.257	-0.110	0.499	0.	0.799	0.151	0.353	-0.074	0.639
MDS-UPDRS II	0.039	0.813	-0.005	0.977	0.009	0.956	-	0.719	-0.061	0.706	**-0.359**	**0.018**
MDS-UPDRS III	0.074	0.648	-0.008	0.960	0.064	0.701	0.	0.943	0.053	0.749	-0.204	0.201
III - tremor	-0.118	0.468	-0.232	0.149	-0.081	0.620	-	0.622	0.115	0.479	-0.080	0.610
III - non-tremor	0.091	0.575	0.113	0.486	0.071	0.664	0.	0.808	-0.017	0.917	-0.118	0.451
MDS-UPDRS IV	0.184	0.257	0.094	0.563	-0.089	0.584	-	0.144	-0.065	0.689	0.015	0.926
TUG	-0.011	0.947	0.163	0.320	0.119	0.469	**0.**	**0.019**	-0.168	0.308	0.024	0.881
BBS	0.241	0.140	-0.075	0.649	-0.064	0.696	-	0.053	-0.221	0.176	0.204	0.195
PPT	**-0.352**	**0.028**	0.106	0.523	0.080	0.630	-	0.522	-0.105	0.523	0.106	0.506
PPT_{rh}	**-0.450**	**0.004**	0.011	0.949	0.108	0.514	-	0.938	-0.097	0.558	0.021	0.897

r_s Spearman's rank correlation coefficient rho, *MDS-UPDRS* Movement Disorder Society Unified Parkinson's Disease Rating Scale Parts I-IV, *TUG* Timed Up and Go, *BBS* Berg Balance Scale, *PPT* Perdue Pegboard Test, PPT_{rh} Perdue Pegboard Test - right hand, *PT* physiotherapy, *OT* occupational therapy, *SLT* speech and language therapy, LED_{diff} Difference in Levodopa-equivalent dose from baseline to discharge. N=40 for MDS-UPDRS scales I, II, IV, n=39 for MDS-UPDRS III, BBS, PPT, PPT_{rh}. Significant values are marked in bold.

Table 4. Spearman's Rank Correlation of Baseline Parameters and Multimodal Complex Treatment Hours with Motor Variables.

Baseline parameters	MDS-UPDRS I		MDS-UPDRS II		MDS-UPDRS III		MDS-UPDRS III- Tremor		MDS-UPDRS III - Nontremor	
	r_s	*p*	r_s	*p*	r_s	*p*	r_s	*p*	r_s	*p*
Age	0.094	0.532	**0.309**	**0.034**	**0.382**	**0.008**	0.178	0.231	**0.366**	**0.011**
Age at Diagnosis	-0.072	0.632	0.118	0.430	**0.299**	**0.041**	0.181	0.223	**0.290**	**0.048**
Disease duration	0.155	0.299	**0.304**	**0.038**	0.159	0.286	-0.007	0.961	0.139	0.353
LED	0.036	0.812	0.130	0.383	0.005	0.973	-0.190	0.202	0.020	0.894
MDS-UPDRS I	-	-	**0.501**	**0.000**	0.141	0.343	-0.104	0.487	0.192	0.195
MDS-UPDRS II	**0.501**	**0.000**	-	-	**0.620**	**0.000**	0.257	0.081	**0.613**	**0.000**
MDS-UPDRS III	0.141	0.343	**0.620**	**0.000**	-	-	**0.497**	**0.000**	**0.952**	**0.000**
MDS-UPDRS IV	0.238	0.107	0.121	0.419	-0.047	0.755	-0.168	0.258	-0.027	0.855
TUG, n=45	**0.295**	**0.049**	**0.677**	**0.000**	**0.483**	**0.001**	0.220	0.147	**0.491**	**0.001**
BBS, n=44	**-0.481**	**0.001**	**-0.597**	**0.000**	**-0.417**	**0.005**	-0.029	0.851	**-0.483**	**0.000**
PPT, n=44	-0.103	0.507	**-0.514**	**0.000**	**-0.591**	**0.000**	**-0.324**	**0.032**	**-0.542**	**0.000**
BDI-II, n=46	**0.527**	**0.000**	0.055	0.718	-0.068	0.652	-0.095	0.528	-0.067	0.660
HAMD-17, n=46	**0.570**	**0.000**	0.211	0.160	-0.005	0.972	-0.080	0.598	-0.011	0.941
AES, n=46	0.264	0.076	**0.312**	**0.035**	**0.301**	**0.042**	-0.177	0.238	**0.385**	**0.008**
MoCA	-0.252	0.088	-0.234	0.113	**-0.403**	**0.005**	0.047	0.756	**-0.432**	**0.002**
PT, n=44	-0.215	0.161	-0.001	0.994	-0.229	0.135	-0.051	0.744	-0.219	0.152
Exercise, n=44	**-0.396**	**0.008**	**-0.436**	**0.003**	**-0.415**	**0.005**	0.172	0.263	**-0.521**	**0.000**
Physical Therapy, n=44	**-0.380**	**0.011**	**-0.499**	**0.001**	**-0.334**	**0.027**	0.134	0.387	**-0.444**	**0.003**
OT, n=44	-0.068	0.658	-0.135	0.381	**-0.304**	**0.045**	-0.163	0.290	**-0.302**	**0.047**

Table S4. *Cont.*

Baseline parameters	MDS-UPDRS IV		TUG		BBS		PPT	
	r_s	*p*	r_s	*p*	r_s	*p*	r_s	*p*
Age	-0.167	0.261	**0.392**[e]	**0.008**	-0.248[d]	0.105	**-0.614**[d]	**0.000**
Age at Diagnosis	**-0.420**	**0.003**	**0.310**[e]	**0.038**	-0.129[d]	0.405	**-0.477**[d]	**0.001**
Disease duration	**0.512**	**0.000**	0.045[e]	0.768	-0.115[d]	0.457	-0.239[d]	0.118
LED	**0.465**	**0.001**	-0.020[e]	0.898	-0.132[d]	0.393	-0.048[d]	0.758
MDS-UPDRS I	0.238	0.107	0.295[e]	0.049	**-0.481**[d]	**0.001**	-0.103[d]	0.507
MDS-UPDRS II	0.121	0.419	**0.677**[e]	**0.000**	**-0.597**[d]	**0.000**	**-0.514**[d]	**0.000**
MDS-UPDRS III	-0.047	0.755	**0.483**[e]	**0.001**	**-0.417**[d]	**0.005**	**-0.519**[d]	**0.000**
MDS-UPDRS IV	-	-	-0.135[e]	0.376	-0.112[d]	0.469	0.033[d]	0.831
TUG, n=45	-0.135	0.376	-	-	**-0.709**[d]	**0.000**	**-0.626**[c]	**0.000**
BBS, n=44	-0.112	0.469	**-0.709**[d]	**0.000**	-	-	**0.464**[b]	**0.002**
PPT, n=44	0.033	0.831	**-0.626**[c]	**0.000**	**0.464**[b]	**0.002**	-	-
BDI-II, n=46	0.122	0.421	-0.024[e]	0.878	-0.127[d]	0.412	**0.337**[d]	**0.026**
HAMD-17, n=46	0.161	0.286	0.065[e]	0.672	0.039[d]	0.803	0.236[d]	0.122
AES, n=46	-0.020	0.890	**0.343**[e]	**0.021**	**-0.430**[d]	**0.004**	**-0.346**[d]	**0.021**
MoCA	-0.027	0.856	-0.197[e]	0.195	0.266[d]	0.081	**0.391**[d]	**0.009**
PT, n=44	-0.130	0.400	-0.210[b]	0.182	0.111[a]	0.491	**0.362**[a]	**0.020**
Exercise, n=44	-0.240	0.116	**-0.494**[b]	**0.001**	**0.485**[a]	**0.001**	**0.403**[a]	**0.009**
Physical Therapy, n=44	0.011	0.946	**-0.396**[b]	**0.010**	0.248[a]	0.117	0.283[a]	0.073
OT, n=44	**0.436**	**0.003**	**-0.361**[b]	**0.019**	0.203[a]	0.202	0.205[a]	0.198

r_s Spearman's rank correlation coefficient rho, *MDS-UPDRS* Movement Disorder Society Unified Parkinson's Disease Rating Scale Parts I-IV, *TUG* Timed Up and Go, *BBS* Berg Balance Scale, *PPT* Perdue Pegboard Test, *PT* physiotherapy, *OT* occupational therapy, *LED* levodopa equivalent dose, *BDI-II* revised Beck Depression Inventory, *HAMD* Hamilton Rating Scale for Depression, *AES* Apathy Evaluation Scale, *MoCA* Montreal Cognitive Assessment, if not indicated otherwise n=47, [a] n=41, [b] n=42, [c] n=43, [d] n=44, [e] n=45, significant values are marked in bold, correlation coefficients > 0.5 are underlined.

Table S5. Predictors of Treatment Response at Discharge.

Response measure	Predictor	Predictor categories	OR	CI		RR	CI		p- value
				LB	UB		LB	UB	
MDS-UPDRS I	Age [y]	<70/≥70	0.94	0.24	3.74	0.99	0.69	1.40	1.000
	Sex	male/female	1.07	0.27	4.25	1.02	0.71	1.45	1.000
	H&Y	<3/≥3	0.57	0.14	2.34	0.87	0.61	1.23	0.501
	Disease duration [y]	<8/≥8	2.25	0.55	9.25	1.23	0.86	1.76	0.310
	LED [mg]	<595/≥595	2.25	0.55	9.25	1.23	0.86	1.76	0.310
	MDS-UPDRS I	<11/≥11	0.44	0.11	1.76	0.80	0.54	1.19	0.295
	MDS-UPDRS II	<13/≥13	1.83	0.41	8.20	1.16	0.82	1.62	0.494
	MDS-UPDRS III	<33/≥33	3.97	0.74	21.35	1.35	0.97	1.88	0.154
	MDS-UPDRS IV	<5/≥5	0.83	0.21	3.29	0.96	0.67	1.36	1.000
	PDQ-39	<30/≥30	1.59	0.40	6.41	1.13	0.77	1.67	0.719
	BDI-II	<14/≥14	1.26	0.30	5.30	1.06	0.72	1.57	1.000
	AES	<11/≥11	1.26	0.30	5.30	1.06	0.72	1.57	1.000
	MoCA	≥26/<26	1.21	0.26	5.56	1.05	0.73	1.52	1.000
	PT [h]	≤4.5/>4.5	1.06	0.27	4.19	1.02	0.72	1.44	1.000
	Exercise [h]	<4.5/≥4.5	0.93	0.24	3.70	0.98	0.69	1.40	1.000
	OT [h]	≤3.5/>3.5	1.54	0.39	6.12	1.12	0.78	1.60	0.728
	SLT [h]	≤2.5/>2.5	1.98	0.48	8.13	1.19	0.83	1.69	0.488
	LED increase	no/yes	1.21	0.26	5.56	1.05	0.73	1.51	1.000
MDS-UPDRS II	Age [y]	<70/≥70	1.94	0.50	7.49	1.20	0.82	1.77	0.497
	Sex	male/female	0.87	0.23	3.34	0.96	0.66	1.39	1.000
	H&Y	<3/≥3	1.21	0.32	4.61	1.06	0.73	1.54	1.000
	Disease duration [y]	<8/≥8	4.75	1.07	21.14	1.51	1.01	2.27	0.045
	LED [mg]	<595/≥595	4.75	1.07	21.14	1.51	1.01	2.27	0.045
	MDS-UPDRS I	<11/≥11	0.88	0.23	3.43	0.97	0.66	1.42	1.000
	MDS-UPDRS II	<13/≥13	2.17	0.49	9.60	1.22	0.85	1.74	0.484
	MDS-UPDRS III	<33/≥33	1.44	0.36	5.84	1.11	0.77	1.60	0.735
	MDS-UPDRS IV	<5/≥5	0.67	0.17	2.57	0.90	0.62	1.30	0.736
	PDQ-39	<30/≥30	3.42	0.86	13.67	1.45	0.91	2.31	0.092
	BDI-II	<14/≥14	1.75	0.44	6.98	1.18	0.76	1.83	0.482
	AES	<11/≥11	1.75	0.44	6.98	1.18	0.76	1.83	0.482
	MoCA	≥26/<26	0.49	0.12	1.98	0.80	0.50	1.29	0.460
	PT [h]	≤4.5/>4.5	2.13	0.53	8.58	1.23	0.85	1.78	0.327
	Exercise [h]	<4.5/≥4.5	1.15	0.30	4.44	1.04	0.72	1.51	1.000
	OT [h]	≤3.5/>3.5	0.28	0.06	1.21	0.72	0.49	1.04	0.099
	SLT [h]	≤2.5/>2.5	0.59	0.15	2.27	0.86	0.59	1.26	0.510
	LED increase	no/yes	0.29	0.07	1.20	0.67	0.40	1.15	0.137
MDS-UPDRS III	Age [y]	<70/≥70	1.25	0.36	4.32	1.09	0.68	1.73	0.761
	Sex	male/female	2.19	0.62	7.70	1.35	0.82	2.21	0.341
	H&Y	<3/≥3	0.36	0.10	1.34	0.70	0.44	1.11	0.206
	Disease duration [y]	<8/≥8	0.72	0.21	2.50	0.89	0.56	1.41	0.755
	LED [mg]	<595/≥595	1.08	0.31	3.71	1.03	0.65	1.63	1.000
	MDS-UPDRS I	<11/≥11	1.15	0.32	4.08	1.05	0.66	1.67	1.000
	MDS-UPDRS II	<13/≥13	0.98	0.27	3.52	0.99	0.62	1.60	1.000
	MDS-UPDRS III	<33/≥33	**0.13**	0.03	0.52	0.44	0.22	0.85	**0.004**
	MDS-UPDRS IV	<5/≥5	1.08	0.31	3.71	1.03	0.65	1.63	1.000
	PDQ-39	<30/≥30	1.02	0.28	3.66	1.01	0.63	1.62	1.000
	BDI-II	<14/≥14	0.57	0.14	2.24	0.82	0.52	1.29	0.512
	AES	<11/≥11	0.91	0.24	3.42	0.97	0.60	1.57	1.000
	MoCA	≥26/<26	0.23	0.06	0.90	0.52	0.26	1.08	0.043
	PT [h]	≤4.5/>4.5	1.80	0.51	6.34	1.24	0.78	1.96	0.528
	Exercise [h]	<4.5/≥4.5	1.03	0.30	3.57	1.01	0.64	1.60	1.000
	OT [h]	≤3.5/>3.5	0.56	0.16	1.97	0.81	0.51	1.28	0.528

	SLT [h]	≤2.5/>2.5	0.62	0.18	2.16	0.84	0.53	1.34	0.537
	LED increase	no/yes	0.93	0.24	3.54	0.97	0.58	1.62	1.000
MDS-UPDRS IV	Age [y]	<70/≥70	1.27	0.38	4.29	1.11	0.66	1.85	0.763
	Sex	male/female	1.02	0.30	3.48	1.01	0.60	1.68	1.000
	H&Y	<3/≥3	0.59	0.17	2.01	0.80	0.48	1.33	0.537
	Disease duration [y]	<8/≥8	0.33	0.09	1.18	0.64	0.37	1.08	0.124
	LED [mg]	<595/≥595	0.74	0.22	2.49	0.88	0.53	1.46	0.760
	MDS-UPDRS I	<11/≥11	0.47	0.14	1.64	0.72	0.40	1.28	0.344
	MDS-UPDRS II	<13/≥13	2.04	0.56	7.49	1.33	0.81	2.17	0.348
	MDS-UPDRS III	<33/≥33	1.57	0.45	5.53	1.20	0.73	1.98	0.541
	MDS-UPDRS IV	<5/≥5	**0.13**	0.03	0.54	0.45	0.25	0.81	**0.005**
	PDQ-39	<30/≥30	1.13	0.32	3.95	1.05	0.62	1.80	1.000
	BDI-II	<14/≥14	2.53	0.69	9.36	1.53	0.79	2.96	0.198
	AES	<11/≥11	1.64	0.45	5.94	1.24	0.68	2.25	0.521
	MoCA	≥26/<26	1.22	0.32	4.63	1.09	0.64	1.85	1.000
	PT [h]	≤4.5/>4.5	2.55	0.72	8.96	1.46	0.88	2.45	0.216
	Exercise [h]	<4.5/≥4.5	2.17	0.62	7.60	1.37	0.83	2.26	0.351
	OT [h]	≤3.5/>3.5	0.39	0.11	1.38	0.68	0.41	1.14	0.216
	SLT [h]	≤2.5/>2.5	0.63	0.19	2.13	0.82	0.49	1.38	0.543
	LED increase	no/yes	1.97	0.50	7.82	1.30	0.79	2.13	0.503
TUG, n=42	Age [y]	<70/≥70	0.75	0.21	2.73	0.91	0.59	1.39	0.750
	Sex	male/female	1.00	0.27	3.66	1.00	0.65	1.54	1.000
	H&Y	<3/≥3	0.87	0.24	3.15	0.95	0.62	1.46	1.000
	Disease duration [y]	<8/≥8	1.00	0.28	3.61	1.00	0.65	1.53	1.000
	LED [mg]	<595/≥595	0.48	0.13	1.81	0.79	0.51	1.21	0.338
	MDS-UPDRS I	<11/≥11	0.86	0.24	3.17	0.95	0.61	1.48	1.000
	MDS-UPDRS II	<13/≥13	0.74	0.20	2.75	0.90	0.57	1.43	0.742
	MDS-UPDRS III	<33/≥33	1.35	0.36	5.08	1.10	0.72	1.69	0.747
	MDS-UPDRS IV	<5/≥5	0.75	0.21	2.73	0.91	0.59	1.39	0.750
	PDQ-39	<30/≥30	0.62	0.16	2.47	0.86	0.56	1.31	0.734
	BDI-II	<14/≥14	0.26	0.05	1.38	0.69	0.47	1.02	0.159
	AES	<11/≥11	1.39	0.35	5.45	1.12	0.68	1.84	0.729
	MoCA	≥26/<26	1.00	0.24	4.14	1.00	0.62	1.61	1.000
	PT [h]	≤4.5/>4.5	1.33	0.37	4.85	1.10	0.72	1.69	0.750
	Exercise [h]	<4.5/≥4.5	1.00	0.27	3.66	1.00	0.65	1.54	1.000
	OT [h]	≤3.5/>3.5	4.50	1.12	18.13	1.64	1.01	2.65	0.049
	SLT [h]	≤2.5/>2.5	0.87	0.24	3.13	0.95	0.62	1.47	1.000
	LED increase	no/yes	1.18	0.29	4.83	1.06	0.68	1.66	1.000
BBS, n=42	Age [y]	<70/≥70	0.56	0.16	1.89	0.76	0.42	1.35	0.374
	Sex	male/female	0.80	0.24	2.73	0.90	0.51	1.60	0.764
	H&Y	<3/≥3	**0.12**	0.03	0.49	**0.39**	0.20	0.75	**0.002**
	Disease duration [y]	<8/≥8	1.00	0.30	3.36	1.00	0.56	1.78	1.000
	LED [mg]	<595/≥595	1.20	0.36	4.04	1.09	0.61	1.95	1.000
	MDS-UPDRS I	<11/≥11	0.70	0.20	2.41	0.84	0.46	1.55	0.754
	MDS-UPDRS II	<13/≥13	0.86	0.25	2.98	0.93	0.51	1.70	1.000
	MDS-UPDRS III	<33/≥33	0.47	0.13	1.64	0.69	0.36	1.32	0.346
	MDS-UPDRS IV	<5/≥5	1.20	0.36	4.04	1.09	0.61	1.95	1.000
	PDQ-39	<30/≥30	1.43	0.40	5.07	1.19	0.63	2.25	0.749
	BDI-II	<14/≥14	0.92	0.25	3.41	0.96	0.52	1.78	1.000
	AES	<11/≥11	0.58	0.15	2.21	0.78	0.44	1.39	0.514
	MoCA	≥26/<26	0.88	0.23	3.34	0.94	0.49	1.81	1.000
	PT [h]	≤4.5/>4.5	0.83	0.25	2.80	0.92	0.51	1.64	1.000
	Exercise [h]	<4.5/≥4.5	1.25	0.37	4.26	1.11	0.63	1.98	0.764
	OT [h]	≤3.5/>3.5	2.63	0.75	9.13	1.59	0.85	2.97	0.216
	SLT [h]	≤2.5/>2.5	2.68	0.77	9.38	1.59	0.88	2.89	0.137
	LED increase	no/yes	0.70	0.19	2.59	0.84	0.43	1.64	0,741

PPT, n=41	Age [y]	<70/≥70	0.95	0.24	3.81	0.99	0.68	1.43	1.000
	Sex	male/female	3.02	0.72	12.70	1.35	0.89	2.04	0.164
	H&Y	<3/≥3	0.95	0.24	3.81	0.99	0.68	1.43	1.000
	Disease duration [y]	<8/≥8	2.29	0.55	9.52	1.25	0.85	1.83	0.306
	LED [mg]	<595/≥595	2.00	0.48	8.30	1.20	0.83	1.74	0.484
	MDS-UPDRS I	<11/≥11	0.48	0.12	1.96	0.82	0.54	1.23	0.476
	MDS-UPDRS II	<13/≥13	1.17	0.28	4.87	1.04	0.72	1.51	1.000
	MDS-UPDRS III	<33/≥33	2.04	0.45	9.24	1.20	0.84	1.71	0.478
	MDS-UPDRS IV	<5/≥5	0.83	0.21	3.33	0.95	0.66	1.38	1.000
	PDQ-39	<30/≥30	1.67	0.41	6.82	1.15	0.76	1.75	0.491
	BDI-II	<14/≥14	1.33	0.31	5.72	1.08	0.71	1.65	0.719
	AES	<11/≥11	2.29	0.55	9.64	1.28	0.80	2.05	0.280
	MoCA	≥26/<26	0.75	0.18	3.22	0.92	0.61	1.41	0.719
	PT [h]	≤4.5/>4.5	1.53	0.37	6.35	1.12	0.78	1.61	0.726
	Exercise [h]	<4.5/≥4.5	0.48	0.12	1.96	0.82	0.54	1.23	0.476
	OT [h]	≤3.5/>3.5	1.37	0.34	5.49	1.09	0.75	1.58	0.734
	SLT [h]	≤2.5/>2.5	7.77	1.42	42.66	1.65	1.08	2.05	0.015
	LED increase	no/yes	0.44	0.10	1.84	0.78	0.49	1.26	0.280

Predictors of Treatment Response at DC in relation to BL examination. If not labeled otherwise, predictor categories refer to total counts in each score. Depicted are odds ratios and relative risk for dichotomized BL parameter function. If not indicated otherwise n=43. y Years, mg Milligrams, h Hours, OR Odds ratio, RR Relative risk, CI Confidence interval, LB Lower bound, UB Upper bound, H&Y modified Hoehn and Yahr stage, MDS-UPDRS I-IV Movement Disorders Society Unified Parkinson's Disease Rating Scale, Parts I-IV, LED Levodopa equivalent dose, PDQ-39 Parkinson's Disease Questionnaire, BDI-II revised Beck Depression Inventory, AES Apathy Evaluation Scale, MoCA Montreal Cognitive Assessment, PT Physiotherapy, OT Occupational therapy, SLT speech and language therapy, significant values with p<0.01 are printed in bold.
